# Effects of Functional Electrical Stimulation on Attention and Brain Activity in Healthy Participants Using Near-Infrared Spectroscopy: An Interventional Study

**DOI:** 10.7759/cureus.57886

**Published:** 2024-04-09

**Authors:** Nao Yoshihiro, Kazu Amimoto, Shinpei Osaki, Junpei Tanabe, Katsuya Sakai, Yumi Ikeda

**Affiliations:** 1 Department of Occupational Therapy, Kansai University of Health Sciences, Osaka, JPN; 2 Graduate School of Human Health Sciences, Tokyo Metropolitan University, Tokyo, JPN; 3 Department of Physical Therapy, Sendai Seiyo Gakuin College, Miyagi, JPN; 4 Department of Rehabilitation, Kansai Electric Power Hospital, Osaka, JPN; 5 Department of Physical Therapy, Hiroshima Cosmopolitan University, Hiroshima, JPN

**Keywords:** reaction time, posner task, functional near-infrared spectroscopy, spatial attention, functional electrical stimulation

## Abstract

Background

Involuntary limb activation using functional electrical stimulation (FES) can improve unilateral spatial neglect. However, the impact of FES on brain activity related to spatial attention remains unclear. Thus, in this study, we aimed to examine the effects of FES on spatial attention.

Methodology

In this interventional study, 13 healthy right-handed participants were asked to perform the Posner task for six minutes both before and after either FES or sham stimulation during each set, resulting in a total of two sets. FES was applied to the left forearm extensor muscles, with a frequency of 25 Hz, a pulse width of 100 μs, and the intensity adjusted to reach the motor threshold. Both the energization and pause times were set to five seconds. The Posner task was used to measure reaction time to a target appearing on a computer screen. Brain activity, indicated by oxygenated hemoglobin values, was measured using near-infrared spectroscopy with 24 probes according to the International 10-20 system method.

Results

In the left hemisphere, oxygenated hemoglobin values in the premotor and supplementary motor areas, primary somatosensory cortex, and somatosensory association areas were significantly higher after FES than after sham stimulation. In the right hemisphere, oxygenated hemoglobin values were significantly increased in the premotor, primary, and supplementary motor areas; in the supramarginal gyrus; and in the somatosensory association areas after FES. Reaction times in the Posner task did not differ significantly between the FES and sham conditions.

Conclusions

Collectively, these results suggest that FES of the upper limbs can activate the ventral pathway of the visual attention network and improve stimulus-driven attention. Activation of stimulus-driven attentional function could potentially contribute to symptom improvement in patients with unilateral spatial neglect.

## Introduction

Unilateral spatial neglect (USN) frequently occurs after cerebrovascular injury. Patients with USN fail to perceive or respond to stimuli in the contralateral space, regardless of sensory or motor disorders [1]. The inferior parietal lobe, superior temporal gyrus, and inferior frontal gyrus play primary roles in USN [2], and lesions involving these regions in the right hemisphere are characteristic of the acute phase of USN [3]. In contrast, lesions are confined to the right temporal lobe in the chronic phase of USN. Recently, the importance of considering localized foci and damage to the white matter fibers (branches II and III of the superior longitudinal fasciculi and the supraorbital fasciculus) in USN has been indicated. Doricchi and Tomaiuolo [4] reported the importance of the superior longitudinal fasciculus and supramarginal gyrus as foci in patients with residual USN symptoms in the chronic phase. The involvement of foci in the neural circuits connecting the temporoparietal region to the lateral aspect of the frontal lobe, including the superior longitudinal fasciculus, in the development of USN has been reported [5].

The spatial attention network is divided into dorsal and ventral pathways [6]. The dorsal pathway extends from the visual cortex of the occipital lobe through the inferior parietal sulcus to the frontal eye fields. It responds to cue stimuli by spontaneously directing attention to a location (goal-directed attention). In contrast, the ventral pathway leads to the ventral frontal cortex, including the middle frontal gyrus, via the temporoparietal junction and superior temporal gyrus. It is involved in diverting attention to unexpected stimuli (stimulus-driven attention). The interactions between these two networks regulate attention, whereas damage to these networks is believed to cause USN. Damage to the ventral pathway leads to functional inactivation of the dorsal pathway, resulting in USN [6]. In patients with USN, spatial attention deficits are more significant when the ventral pathway is damaged than when the dorsal pathway is involved [7]. Therefore, it is important to consider the ventral pathway of the spatial attention network when treating patients with USN.

Active movement of the paralyzed upper limb in the contralesional space significantly reduced neglect symptoms in letter-cancelling and reading tests [8]. Làdavas et al. [9] reported that USN symptoms reduced when another person moved the paralyzed hand in the contralesional space; thus, involuntary limb movements can improve USN. Functional electrical stimulation (FES) devices, which are often used in clinical settings, can reduce USN [10]. Therefore, FES may be beneficial as an intervention for USN because it can be used regardless of the severity of motor paralysis.

Upper limb activation using FES appears to be a readily applicable approach for patients with USN. However, well-founded and practical intervention protocols have not been established because of variability in USN symptoms and stimulation conditions [10,11]. Additionally, electrical stimulation of the upper limbs reportedly activates the bilateral somatosensory cortex [12]. However, the effects on the inferior parietal lobule and other areas related to USN have not been clarified. Here, we hypothesized that FES activates other brain regions, including the supramarginal gyrus and angular gyrus, in addition to the somatosensory and motor cortices. Therefore, in this study, we aimed to clarify brain activity induced by upper limb activation using FES, in healthy participants. Our findings will be applied to a promising method for USN.

## Materials and methods

Participants

The study included 13 healthy, young, right-handed individuals with no history of neurologic or medical conditions (mean age: 26.8 ± 5.1 years; seven men). All participants provided written informed consent to participate in this study. This study was conducted in accordance with the Declaration of Helsinki and approved by the Ethics Committee of the Tokyo Metropolitan University (approval number: 19094).

Modified Posner task

**Figure 1:**
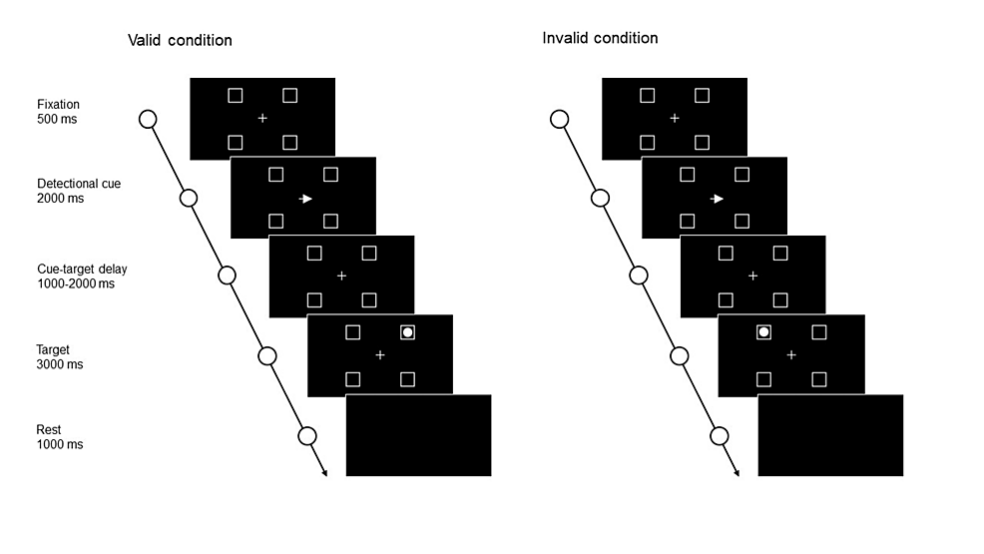
The modified Posner task. The figure shows a representation of the task on the personal computer monitor. In the valid condition, an arrow pointing left or right was presented on the screen as a cue, and the target was then presented on the side corresponding to the arrow’s direction. In the invalid condition, the target was presented in the opposite direction of the cue condition.

A modified Posner task (Figure 1) was used to investigate the effect of FES on attentional function [13]. A computer (GL65-9SD-046JP; Micro-Star International Co., Ltd, New Taipei City, Taiwan) was used to display the task on a 15.6-inch monitor with a refresh rate of 120 Hz. Behavioral responses were recorded using a numeric keypad (TK-TCM011; Elecom Co., Ltd., Osaka, Japan) connected to the computer. A modified Posner task was created using SuperLab 5.0 (Cedrus Corporation, San Pedro, CA, USA). A central fixation cross (fixed viewpoint) and a frame of four squares placed on the left and right sides along the horizontal meridian were presented on the monitor. The width of each square was within a visual angle of 1°, and a circular target was placed at the center of one square. The diameter of each target had a visual angle of 0.3°, and the four surrounding squares were presented at a visual angle of 4.3° away from the fixation point.

After 500 ms of the fixation phase, an arrow cue pointing left or right appeared over the fixation cross for 2,000 ms. Subsequently, after a delay of 1,000-2,000 ms, a circular target appeared in one of the four squares (top left, bottom left, top right, or bottom right) for 3,000 ms. In the valid condition (80% of trials), the target appeared on the side corresponding to the direction of the cue arrow; in the invalid condition (20% of trials), the target appeared on the side opposite to the cue direction. The participants were instructed to detect the target as quickly as possible and to press a key with the right index finger once the target was detected. The reaction times (RTs) of the key presses were recorded. During the task, the participants were seated in a chair placed approximately 50 cm from the monitor. The total duration of the task was six minutes.

Apparatus

The Trio300 system (SE-231; ITO Co., Ltd., Saitama, Japan) was used for FES. The stimulation electrodes were attached to the left extensor digitorum muscle. The stimulation pulse frequency was set at 25 Hz, the pulse width at 100 μs, and the intensity at the level required to reach the motor threshold. The energization and pause times were both set to five seconds.

Functional near-infrared spectroscopy (fNIRS) measurement

**Figure 2:**
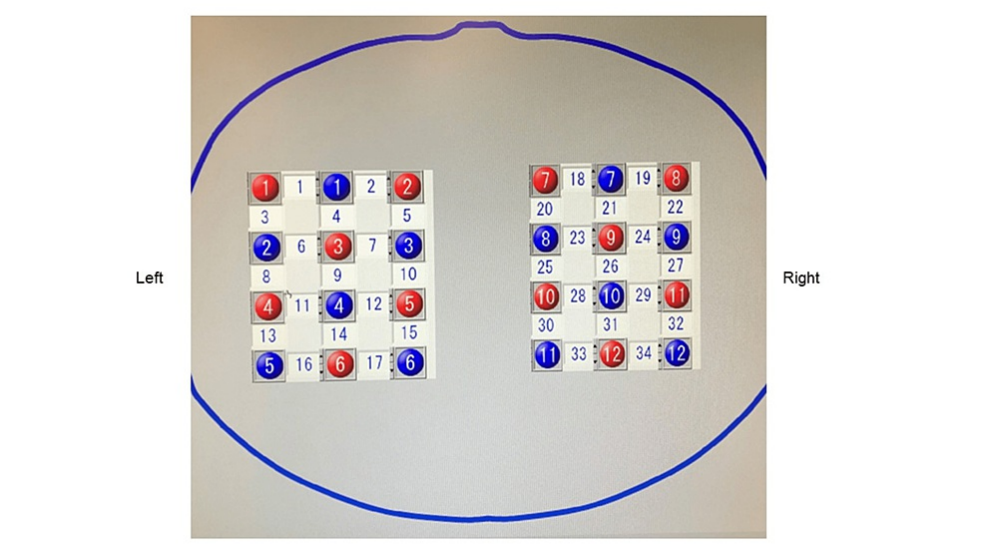
Functional near-infrared spectroscopy (fNIRS) channel layout and the location of the region of interest (ROI). The probe consisted of 34 channels using 12 sources (red) and 12 detectors (blue) with a source-detector distance of 30 mm. The ROIs identified by fNIRS statistical parametric mapping using a three-dimensional digitizer were as follows: the dorsolateral prefrontal cortex (channels 1 and 19), frontal eye fields (channels 2 and 18), premotor and supplementary motor cortices (channels 3-7 and 20-23), the primary somatosensory cortex (channels 8, 27, and 28), the primary motor cortex (channels 9, 10, 12, and 24-26), the supramarginal gyrus (channels 11, 13, 14, 29, 31, 32, and 34), the somatosensory association cortex (channels 15, 17, 30, and 33), and the angular gyrus (channel 16). Channels 1-17 were located in the left hemisphere and channels 18-34 in the right hemisphere.

A near-infrared optical brain function imaging system (LABNIRS; Shimadzu Co., Ltd., Kyoto, Japan) was used to measure brain activity. This continuous wave-type fNIRS system was allowed to warm up for at least 30 minutes before beginning the recordings. The wavelengths of fNIRS were 780, 805, and 850 nm. The probe position was 3 × 4 on each side, and 24 probes (12 sources and 12 detectors) were set for 34 channels (Figure 2).

fNIRS is used to measure oxygenated (oxy-Hb) and deoxygenated (deoxy-Hb) hemoglobin levels based on the modified Beer-Lambert law [14]. However, oxy-Hb values are more sensitive than deoxy-Hb values, and individual differences in changes in deoxy-Hb values have been reported; therefore, oxy-Hb values were primarily analyzed as an indicator of brain activity [15].

The channel layout considered the nearest neighbor area. The probe distance was 30 mm, and the sampling rate was 10 Hz. The nasal root, occipital tubercle, and right and left external auditory meatuses were measured using a whole-head holder according to the International 10-20 system of electrode placement, and their midpoints were defined as Cz for the whole-head holder. The probe position was determined such that it covered the top of the head.

fNIRS analysis

We manually removed oxy-Hb data that contained low signal-to-noise ratios from multiple channels owing to faulty light sources or incorrect detector placement [16], as well as data that contained visually obvious motion artifacts. Moreover, a 0.01-0.10 Hz band-pass filter was applied to the oxy-Hb fNIRS signals [17,18], which was able to remove the artifacts due to physiological activity (i.e., Mayer waves, respiration, and heartbeats) [17].

All channels were marked with 10-20-system landmarks (the nasion, inion, right, left, and preauricular points) and recorded using a three-dimensional digitizer (3 SPACE, FASTRAK; Polhemus Co., Ltd., Colchester, VT, USA) to determine the brain region corresponding to each channel location. All channels were then transformed using NIRS statistical parametric mapping to convert these coordinates into 34 channel positions based on the estimated Montreal Neurological Institute space [19,20]. NIRS statistical parametric mapping was used to generate three-dimensional functional images in the Montreal Neurological Institute space using probabilistic registration with reference to the digitized data and landmark locations of the 10-20 system [18,20].

The regions of interest were the dorsolateral prefrontal cortex (DLPFC), frontal eye fields, premotor and supplementary motor areas, primary motor area, primary somatosensory area, somatosensory association area, supramarginal gyrus, and angular gyrus. The correspondence between the regions of interest and the channels is presented in Table 1.

**Table 1:** Correspondence between ROIs and channels. ROI = region of interest; Ch = channel

Left hemisphere		Right hemisphere	
ROI	Ch	ROI	Ch
Dorsolateral prefrontal cortex	1	Frontal eye fields	18
Frontal eye fields	2	Dorsolateral prefrontal cortex	19
Premotor and supplementary motor cortices	3, 4, 5, 6, 7	Premotor and supplementary motor cortices	20, 21, 22, 23
Primary somatosensory cortex	8	Primary motor cortex	24, 25, 26
Primary motor cortex	9, 10, 12	Primary somatosensory cortex	27, 28
Supramarginal gyrus part of Wernicke’s area	11, 13, 14	Supramarginal gyrus part of Wernicke’s area	29, 31, 32, 34
Somatosensory association cortex	15, 17	Somatosensory association cortex	30, 33
Angular gyrus, part of Wernicke’s area	16		

Procedure

The participants were asked to sit on a chair and remain as still as possible. The measurement protocol consisted of six minutes of the Posner task before stimulation, six minutes of stimulation, and six minutes of the Posner task after stimulation in each set. A one-minute rest period was allowed before and after each task performance. The participants were randomly assigned to perform the task in either of the two orders, that is, (a)(b) or (b)(a), where (a) was the Posner task performed before and after FES, and (b) was the Posner task performed before and after sham stimulation. For sham stimulation, electrodes were applied to the left extensor digitorum muscle, as during FES stimulation, and electrical stimulation was applied for approximately five seconds immediately after the start of the task. The duration of the task was approximately 40 minutes.

Statistical analysis

For the analysis of RTs in the Posner task, a two-way analysis of variance (repeated-measures split-plot) was conducted on RTs. The factors included the presence of a stimulus (FES or sham stimulation) and before and after the stimulus as factors for each target presentation condition (target appearance on the left side in the invalid condition, Lt-invalid; on the left side in the valid condition, Lt-valid; on the right side in the invalid condition, Rt-invalid; on the right side in the valid condition, Rt-valid). Furthermore, for percentage changes in RT (post-stimulus RT - pre-stimulus RT)/pre-stimulus RT × 100 before and after FES and before and after the sham stimulation, we performed paired t-tests for each target presentation condition. If the data did not follow a normal distribution, Wilcoxon’s signed-rank test was used.

Oxy-Hb values were used to analyze brain activity. For each measurement channel, we calculated the mean value during the Posner task for FES and sham stimulation for six minutes. The rate of change between the pre- and post-stimulus values was calculated using the following formula: (Oxy-Hb value during post-stimulus Posner task - Oxy-Hb value during pre-stimulus Posner task)/Oxy-Hb value during pre-stimulus Posner task × 100. The results of the Shapiro-Wilk test indicated that the rate of change did not follow a normal distribution. The Wilcoxon rank-sum test was used to compare brain activity during FES stimulation and sham stimulation. All tests were performed using SPSS for Windows, Version 25.0 (IBM Corp., Armonk, NY, USA), with a significance level of p < 0.05.

## Results

RT

Table 2 shows the RTs in the Posner task. The two-way analysis of variance revealed a significant interaction between the stimulus condition and measurement period in the Rt-valid condition (p = 0.01), with a tendency for post-stimulus RTs to differ between the FES and sham stimulation groups. In the Lt-valid condition, the post-stimulus RTs differed between the FES and sham stimulation groups, although the difference was not statistically significant (p = 0.07). No significant differences were observed in the other target presentation conditions (Lt-invalid and Rt-invalid). The paired t-test and Wilcoxon’s signed-rank test revealed no significant differences in the rate of changes in RT before and after FES or before and after sham stimulation.

**Table 2:** Average reaction time (ms). Pre-sham = Posner task result before sham; Post-sham = Posner task result after sham; FES = functional electrical stimulation; Pre-FES = Posner task result before FES; Post-FES = Posner task result after FES; Lt-invalid = target appearance on the left side in the invalid condition; Lt-valid = target appearance on the left side in the valid condition; Rt-invalid = target appearance on the right side in the invalid condition; Rt-valid = target appearance on the right side in the valid condition

	Lt-invalid	Lt-valid	Rt-invalid	Rt-valid
Pre-sham	383.41 ± 55.65	351.36 ± 38.76	361.45 ± 46.47	352.76 ± 38.32
Post-sham	367.19 ± 51.09	357.05 ± 40.49	374.59 ± 74.17	369.84 ± 51.41
Pre-FES	370.63 ± 61.34	354.36 ± 48.27	358.51 ± 52.80	355.04 ± 40.68
Post-FES	365.01 ± 54.09	346.69 ± 38.20	361.41 ± 40.18	347.51 ± 41.04

Brain activity

**Figure 3:**
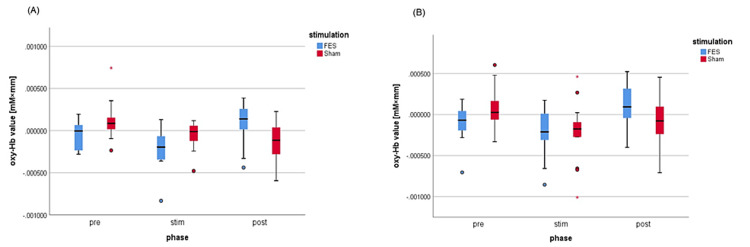
Changes in oxy-Hb values in each period. (A) Premotor and supplementary motor cortices in the right hemisphere. (B) Supramarginal gyrus in the right hemisphere. oxy-Hb = oxygenated hemoglobin; FES = functional electrical stimulation; Pre = oxy-Hb value before stimulation; Stim = oxy-Hb value during stimulation; Post = oxy-Hb value after stimulation

The oxy-Hb values in each channel are presented in Table 3, and changes in oxy-Hb values for each period in regions of interest with significant activity are shown in Figure 3. The percentages of channels excluded owing to insufficient light were as follows: channels 4, 17, and 20 were excluded by 20%; channels 10 and 25 by 7%; and channels 5, 15, and 21 by 23%.

**Table 3:** Oxy-Hb values (10-3 mM × mm). oxy-Hb = oxygenated hemoglobin; Ch = channel; FES = functional electrical stimulation; Pre-FES = before FES; Post-FES = after FES; Pre-sham = before sham; Post-sham = after sham

	Pre-FES	FES	Post-FES	Pre-sham	Sham	Post-sham
Ch 1	0.09 ± 0.20	-0.18 ± 0.32	0.11 ± 0.31	0.06 ± 0.19	-0.14 ± 0.30	-0.01 ± 0.23
Ch 2	-0.05 ± 0.24	-0.12 ± 0.31	0.08 ± 0.29	0.08 ± 0.27	-0.11 ± 0.23	-0.02 ± 0.23
Ch 3	0.06 ± 0.12	-0.20 ± 0.39	0.09 ± 0.38	0.05 ± 0.19	-0.15 ± 0.37	-0.05 ± 0.16
Ch 4	-0.13 ± 0.22	-0.24 ± 0.35	0.18 ± 0.30	-0.04 ± 0.15	-0.20 ± 0.18	0.15 ± 0.20
Ch 5	-0.17 ± 0.38	-0.19 ± 0.20	0.16 ± 0.19	-0.11 ± 0.36	-0.15 ± 0.15	-0.09 ± 0.26
Ch 6	-0.04 ± 0.19	-0.16 ± 0.28	0.05 ± 0.21	-0.01 ± 0.16	-0.12 ± 0.27	-0.08 ± 0.28
Ch 7	-0.09 ± 0.25	-0.14 ± 0.23	0.03 ± 0.18	-0.12 ± 0.23	-0.12 ± 0.17	-0.04 ± 0.32
Ch 8	-0.04 ± 0.13	-0.10 ± 0.38	0.05 ± 0.31	0.07 ± 0.18	-0.18 ± 0.35	-0.15 ± 0.27
Ch 9	-0.12 ± 0.23	-0.22 ± 0.31	0.05 ± 0.22	-0.02 ± 0.15	-0.15 ± 0.24	-0.05 ± 0.31
Ch 10	0.00 ± 0.17	-0.15 ± 0.28	0.18 ± 0.18	-0.06 ± 0.28	-0.04 ± 0.27	-0.16 ± 0.37
Ch 11	-0.06 ± 0.25	-0.21 ± 0.31	0.11 ± 0.30	0.04 ± 0.17	-0.22 ± 0.35	-0.22 ± 0.32
Ch 12	-0.05 ± 0.24	-0.20 ± 0.28	0.02 ± 0.21	0.05 ± 0.23	-0.16 ± 0.35	-0.11 ± 0.31
Ch 13	-0.02 ± 0.21	-0.13 ± 0.37	0.08 ± 0.39	0.02 ± 0.20	-0.20 ± 0.38	-0.31 ± 0.37
Ch 14	-0.15 ± 0.23	-0.22 ± 0.34	0.01 ± 0.30	0.01 ± 0.18	-0.10 ± 0.28	-0.11 ± 0.33
Ch 15	-0.09 ± 0.21	-0.24 ± 0.38	0.04 ± 0.22	0.06 ± 0.14	-0.14 ± 0.38	-0.06 ± 0.29
Ch 16	-0.16 ± 0.32	-0.19 ± 0.29	0.01 ± 0.21	0.07 ± 0.23	-0.13 ± 0.27	-0.07 ± 0.30
Ch 17	-0.10 ± 0.28	-0.36 ± 0.23	0.06 ± 0.12	0.04 ± 0.15	-0.13 ± 0.29	0.02 ± 0.32
Ch 18	0.01 ± 0.12	-0.07 ± 0.17	0.03 ± 0.26	0.10 ± 0.20	-0.12 ± 0.21	-0.03 ± 0.25
Ch 19	0.00 ± 0.17	-0.03 ± 0.25	0.04 ± 0.32	0.12 ± 0.22	-0.03 ± 0.24	-0.22 ± 0.33
Ch 20	-0.10 ± 0.27	-0.16 ± 0.17	0.10 ± 0.23	0.11 ± 0.24	-0.11 ± 0.18	-0.09 ± 0.17
Ch 21	-0.06 ± 0.35	-0.23 ± 0.25	0.25 ± 0.22	0.04 ± 0.29	-0.05 ± 0.27	0.13 ± 0.40
Ch 22	0.04 ± 0.16	-0.05 ± 0.17	0.13 ± 0.23	0.17 ± 0.20	-0.14 ± 0.26	-0.06 ± 0.34
Ch 23	-0.05 ± 0.16	-0.21 ± 0.25	0.09 ± 0.24	0.13 ± 0.24	-0.07 ± 0.17	-0.14 ± 0.23
Ch 24	0.02 ± 0.09	-0.15 ± 0.30	0.07 ± 0.20	0.14 ± 0.20	-0.14 ± 0.28	-0.11 ± 0.28
Ch 25	-0.15 ± 0.19	-0.20 ± 0.31	0.13 ± 0.21	0.12 ± 0.22	-0.19 ± 0.27	0.00 ± 0.22
Ch 26	-0.04 ± 0.13	-0.28 ± 0.40	0.12 ± 0.22	0.16 ± 0.28	-0.23 ± 0.37	-0.13 ± 0.39
Ch 27	-0.02 ± 0.17	-0.08 ± 0.23	0.09 ± 0.18	0.11 ± 0.23	-0.13 ± 0.28	-0.13 ± 0.23
Ch 28	-0.13 ± 0.19	-0.26 ± 0.30	0.11 ± 0.15	0.08 ± 0.23	-0.20 ± 0.31	0.01 ± 0.29
Ch 29	-0.10 ± 0.23	-0.23 ± 0.30	0.14 ± 0.26	0.10 ± 0.26	-0.22 ± 0.39	-0.09 ± 0.30
Ch 30	-0.12 ± 0.25	-0.16 ± 0.30	0.07 ± 0.17	0.15 ± 0.31	-0.10 ± 0.42	-0.05 ± 0.27
Ch 31	-0.16 ± 0.20	-0.26 ± 0.24	0.08 ± 0.16	0.06 ± 0.21	-0.20 ± 0.32	-0.04 ± 0.35
Ch 32	-0.18 ± 0.29	-0.13 ± 0.36	0.07 ± 0.32	-0.15 ± 0.18	-0.18 ± 0.43	0.11 ± 0.34
Ch 33	-0.15 ± 0.27	-0.18 ± 0.41	0.11 ± 0.25	0.17 ± 0.28	-0.22 ± 0.38	0.03 ± 0.30
Ch 34	-0.22 ± 0.25	-0.16 ± 0.21	0.02 ± 0.17	0.03 ± 0.19	-0.23 ± 0.35	-0.02 ± 0.33

In the left hemisphere, oxy-Hb values were significantly higher in the premotor and supplementary motor cortices, primary somatosensory cortex, and somatosensory association cortex (channels 5, 8, and 15) after FES than after sham stimulation (p < 0.05) (Table 4).

**Table 4:** Percentage changes in Oxy-Hb values from pre- to post-stimulation (or sham). oxy-Hb = oxygenated hemoglobin; Ch = channel; FES, functional electrical stimulation Sham: (Oxy-Hb value during post-sham Posner task - Oxy-Hb value during pre-sham Posner task)/Oxy-Hb value during pre-sham Posner task × 100 FES: (Oxy-Hb value during post-FES Posner task - Oxy-Hb value during pre-FES Posner task)/Oxy-Hb value during pre-FES Posner task × 100

	Sham	FES	P-value (×10^－^^1^)
Ch 5	23.07 ± 96.08	38.48 ± 101.29	0.34
Ch 8	-23.09 ± 96.07	38.47 ± 101.27	0.08
Ch 15	-69.24 ± 96.07	38.47 ± 75.11	0.05
Ch 19	-69.25 ± 103.78	-7.69 ± 75.11	0.04
Ch 20	-38.47 ± 96.06	38.47 ± 96.07	0.19
Ch 21	-53.83 ± 103.78	7.72 ± 87.71	0.23
Ch 23	-69.24 ± 101.26	23.09 ± 75.10	0.08
Ch 24	-69.24 ± 103.78	-7.69 ± 75.10	0.23
Ch 25	-69.23 ± 87.70	53.86 ± 75.11	0.04
Ch 26	-53.86 ± 96.07	38.47 ± 87.70	0.09
Ch 29	-23.09 ± 96.61	38.48 ± 101.27	0.03
Ch 31	-23.08 ± 75.10	69.24 ± 101.26	0.05
Ch 32	-38.47 ± 96.07	38.47 ± 96.06	0.28
Ch 33	-53.84 ± 87.70	53.86 ± 87.70	0.04
Ch 34	-38.46 ± 87.70	53.85 ± 96.07	0.19

In the right hemisphere, oxy-Hb values were significantly increased in the premotor and supplementary motor cortices, primary cortex, supramarginal gyrus, and somatosensory association cortex (channels 20, 21, 23, 25, 26, 29, and 31-34; p < 0.05) (Table 4). Additionally, oxy-Hb values in the right DLPFC and primary motor cortex were significantly lower after sham stimulation than after FES (channels 19 and 24; p < 0.05) (Table 4).

## Discussion

In this study, we investigated the effects of involuntary limb activation by FES on spatial attention using the Posner task. The results showed no significant differences between FES and sham stimulation regarding RTs in the Posner task, while different effects were observed for brain activity.

RT

Sensory stimulation of the unilateral posterior neck in healthy participants can shift the self-centered coordinate system to the stimulated side [21]. In the present study, FES of the left upper limb was expected to induce brain activity in the right hemisphere and improve responses to stimuli in the left space. However, no significant differences were observed in RTs in the Posner task between the unstimulated and stimulated conditions because it was difficult to detect such differences in these healthy young participants. The mean RT in the Posner task in this study was shorter than that reported in a previous study also conducted among healthy participants [7,22]. Thus, the data suggested a near-ceiling effect in the present study.

Additionally, a previous study on patients with left USN reported that electrical stimulation of the left hand did not show immediate effects when compared with sham stimulation, while improved performance was observed in cancellation and reading tasks after one month of repeated stimulation [23]. Herein, we examined the immediate effects of a six-minute FES intervention. Therefore, long-term effects should be examined in the future.

Brain activity

The results of this study revealed activation of the primary somatosensory cortex, somatosensory association cortex, and premotor and supplementary motor cortices in the left hemisphere. In the right hemisphere, the premotor and supplementary motor cortices, primary motor cortex, supramarginal gyrus, and somatosensory association area were activated after FES; however, this was not seen after sham stimulation. Additionally, the lateral part of the left premotor and supplementary motor areas and the right DLPFC showed reduced activity in the sham condition compared with those in the FES condition.

Sasaki et al. [12] applied electrical stimulation to the extensor and flexor muscles of the upper limbs and fingers of patients with hemiplegia at a frequency of 18-36 Hz and had participants perform flexion and extension exercises of the fingers for 30-120 minutes per day for 12 weeks. After four weeks of electrical stimulation, increased brain activation was widespread, including in the bilateral somatosensory areas. After continued treatment for 8-12 weeks, the increased activation was limited to the somatosensory areas. The contralateral central posterior gyrus (somatosensory cortex) was activated immediately after electrical stimulation of the hand and extensor muscles at a frequency of 50 Hz and pulse width of 200 μs for 60 hours over three weeks [24]. Our results support those of previous studies, indicating that somatosensory activation persists after FES.

Shin et al. [25] performed electrical stimulation of the extensor digitorum muscle twice daily for 10 weeks at a frequency of 35 Hz, pulse width of 200 μs, 10-20 mA, and an intensity above the motor threshold. Functional magnetic resonance imaging results demonstrated that FES activated the contralateral somatosensory cortex and supplementary motor area while suppressing the ipsilateral premotor and supplementary motor areas. Additionally, simple repetitive voluntary movements, but not FES, reportedly widely activate peripheral regions of the cerebral cortex, such as the supplementary motor and premotor areas [12]. In the present study, the left hand was stimulated by FES, and the participants used the index finger of their right hand to press a key during the Posner task. The activation of the premotor and supplementary motor cortices in the right hemisphere may have persisted after FES.

In contrast, activation of the same cortices in the left hemisphere after FES may have been related to movement of the right hand. The cortices were significantly activated after FES, although right-handed movements were also performed in response to the Posner task in the sham condition. This suggested that FES of the left upper limb affected the premotor and somatosensory cortices in both hemispheres, which contradicts the findings of previous studies suggesting that FES suppresses the ipsilateral premotor and supplementary motor cortices. However, bilateral activation has been reportedly observed in the early stages of recovery, and subsequent functional recovery is focused on the activation of the contralateral hemisphere [26]; thus, the bilateral premotor and supplementary motor areas might have been activated immediately after FES in this study. The premotor and supplementary motor areas are involved in the neglect of extrapersonal spaces [27]. We could not examine the effects of FES on the attention function in the distal space. Hence, the effects of FES on attentional tasks in the extrapersonal space should be examined in future studies.

The DLPFC is involved in attentional and executive functions. It is important to select and direct attention to relevant information from various sensory sources, as well as from working memory, filtering non-relevant stimuli and focusing attention [28]. The right DLPFC is particularly involved in exploratory attention [29]. The present results demonstrated that the activity of the right DLPFC was reduced after sham stimulation, but not after FES. RTs in the Posner task showed a trend toward being delayed after sham stimulation, although the difference was not significant. These results suggested that FES of the left upper limb may prevent the inhibition of right DLPFC activity.

The supramarginal gyrus plays an essential role in the recognition and use of objects through sensory and visual information, and damage to the supramarginal gyrus is associated with self-centered neglect of personal space [5]. Activation of the supramarginal gyrus by FES has not been previously reported. However, our study differed from previous studies in that the Posner task was performed before and after FES. According to Corbetta and Shulman [6], the dorsal pathway for goal-directed attention is bilateral, whereas the ventral pathway for stimulus-driven attention exhibits right hemispheric dominance. Nevertheless, it is difficult to make direct comparisons between these studies, as Corbetta and Shulman’s study [6] focused on participants with cerebrovascular diseases, whereas our study focused on healthy participants. However, FES may activate the ventral pathway of the attentional network more than the dorsal pathway because FES of the left upper limb activates the intrinsic receptive map in the right parietal lobe [10].

FES may enhance brain activity induced by tasks performed simultaneously. In a previous study, the effects of FES on involuntary movements of the upper limbs varied depending on the content of the tasks and the space in which the tasks were performed [30]. The results of this study suggest that the Posner task may activate the supramarginal gyrus, and FES may further enhance this activation. Activation of the supramarginal gyrus is essential for patients with USN in the chronic phase [4]; thus, the combination of FES and the Posner task, which requires stimulus-driven attention, may lead to an improvement in USN symptoms.

Limitations

First, fNIRS results were used as the primary outcome in this study. However, fNIRS usage is limited because it cannot provide information regarding subcortical activity. Thus, future studies are needed to simultaneously investigate the use of fNIRS and functional magnetic resonance imaging. Second, this study was conducted in healthy young adults. Therefore, it is necessary to expand the age range of participants and consider patients with USN in future studies.

## Conclusions

We investigated the effects of FES on spatial attention in healthy participants using fNIRS. The results demonstrated that FES activated the supramarginal gyrus and somatosensory motor and supplementary motor cortices. Thus, FES of the hand extensor muscles may activate the ventral pathway of the visual attention network and improve stimulus-driven attentional function. Activation of stimulus-driven attentional function could then lead to symptom improvement in patients with USN.
